# Distribution of bacteriologically positive and bacteriologically negative pulmonary tuberculosis in Northwest China: spatiotemporal analysis

**DOI:** 10.1038/s41598-022-10675-1

**Published:** 2022-04-27

**Authors:** Hualin Jiang, Xiaolu Sun, Zhongqiu Hua, Haini Liu, Yi Cao, Dan Ren, Xin Qi, Tianhua Zhang, Shaoru Zhang

**Affiliations:** 1 Health Science Centre, Xi’an Jiaotong University, Xi’an, 710061 China; 2 Shaanxi Provincial Institute for Tuberculosis Control and Prevention, Xi’an, 710048 China; 3 Wuxi Early Intervention Centre for Children With Special Needs, Wuxi, 214000 China; 4 Shangluo University, Shangluo, 726000 China

**Keywords:** Infectious diseases, Tuberculosis

## Abstract

Pulmonary tuberculosis (PTB) is a major health issue in Northwest China. Most previous studies on the spatiotemporal patterns of PTB considered all PTB cases as a whole; they did not distinguish notified bacteriologically positive PTB (BP-PTB) and notified bacteriologically negative PTB (BN-PTB). Thus, the spatiotemporal characteristics of notified BP-PTB and BN-PTB are still unclear. A retrospective county-level spatial epidemiological study (2011–2018) was conducted in Shaanxi, Northwest China. In total, 44,894 BP-PTB cases were notified, with an average annual incidence rate of 14.80 per 100,000 persons between 2011 and 2018. Global Moran’s *I *values for notified BP-PTB ranged from 0.19 to 0.49 (*P* < 0.001). Anselin’s local Moran’s *I *analysis showed that the high–high (HH) cluster for notified BP-PTB incidence was mainly located in the southernmost region. The primary spatiotemporal cluster for notified BP-PTB (*LLR* = 612.52, *RR* = 1.77,* P* < 0.001) occurred in the central region of the Guanzhong Plain in 2011. In total, 116,447 BN-PTB cases were notified, with an average annual incidence rate of 38.38 per 100,000 persons between 2011 and 2018. Global Moran’s *I *values for notified BN-PTB ranged from 0.39 to 0.69 (P < 0.001). The HH clusters of notified BN-PTB were mainly located in the north between 2011 and 2014 and in the south after 2015. The primary spatiotemporal cluster for notified BN-PTB (*LLR* = 1084.59, *RR* = 1.85, *P* < 0.001) occurred in the mountainous areas of the southernmost region from 2014 to 2017. Spatiotemporal clustering of BP-PTB and BN-PTB was detected in the poverty-stricken mountainous areas of Shaanxi, Northwest China. Our study provides evidence for intensifying PTB control activities in these geographical clusters.

## Introduction

Pulmonary tuberculosis (PTB) is a major public health problem, with an estimated 9.87 million new cases worldwide in 2020^1^. China had the second largest burden of new TB cases in the world in 2020, with an estimated 842,000 new cases, accounting for 8.5% of the global incidence^1^. According to the Fifth Chinese Epidemiological Survey in 2010, the prevalence of active PTB and bacteriologically positive (BP-PTB) in West China was 695 per 100,000 persons and 198 per 100,000 persons, respectively, which are higher than those for Central and East China^2^. The northwest region of West China is regarded as a high-risk area for PTB^3^. Thus, the identification of PTB epidemic characteristics in this area and promotion of targeted control is urgent^4,5^.

Spatiotemporal cluster analysis has been applied to detect infectious disease hotspots in recent years^6–8^. The global Moran’s *I* test is used most frequently to assess the presence of an overall area-wide spatial autocorrelation. The local Moran’s *I *is used to detect the location and types of spatial clusters^9^. In addition*,* Kulldorff’s space–time scan statistic is the most commonly employed method to detect spatiotemporal clusters^10^. Due to their different methods of testing, areas identified as hotspots by different methods are not identical. Increasingly, the use of multiple cluster analysis methods is recommended and their overlap is used to identify a truly high-risk area^10^.

Most of the previous studies on the spatiotemporal patterns of PTB only considered all PTB cases as a whole and did not distinguish notified BP-PTB and notified bacteriologically negative PTB (BN-PTB)^11,12^. BP-PTB is the most infectious type of PTB and even contributes to outbreaks in crowds. It includes smear-positive PTB and sputum smear-negative but culture-positive PTB^13^. A few studies have examined the spatiotemporal characteristics of smear-positive PTB^14^. However, they did not analyse the spatiotemporal characteristics of sputum smear-negative but culture-positive PTB. Similar to sputum smear-positive cases, sputum culture-positive cases are treated as confirmed cases and have the same treatment protocol and cure criteria as sputum smear-positive cases. Combining smear-positive PTB and sputum smear-negative but culture-positive PTB to analyse the spatiotemporal nature of notified BP-PTB would facilitate unified resource allocation.

Although BN-PTB is less infectious than BP-PTB, it accounts for 50–70% of all PTB cases^15^. However, it is difficult to diagnose. Additionally, half of BN-PTB cases are predicted to convert to BP-PTB without treatment^16^, which increases the difficulty of ending this epidemic. Therefore, proper diagnosis and early treatment are essential for BN-PTB. Information on the spatiotemporal clustering of notified BN-PTB would facilitate early detection and active case identification in high-risk regions.

Shaanxi is a less-developed area of Northwest China that has a serious PTB epidemic. In 2018, approximately 21,442 PTB cases were reported in Shaanxi, with an overall notification rate of 55.49 cases per 100,000 individuals^17^. An epidemiological characteristics analysis found that notified BP-PTB and notified BN-PTB incidence exhibited spatial and temporal heterogeneity^3^. In the current study, we conducted a case study in Shaanxi to explore the spatiotemporal patterns of notified BP-PTB and notified BN-PTB. Using county-level notification data between 2011 and 2018, we carried out spatial variation pattern analyses, spatial autocorrelation analyses and space–time scans. The aim of this study was to clarify the spatiotemporal distribution characteristics of notified BP-PTB and notified BN-PTB in Northwest China, thus providing evidence to inform interventions and improve PTB control.

## Methods

### Study site

Shaanxi (longitude: 105° 29ʹ to 111° 15' E, latitude: 31° 42' to 39° 35' N) is a province in Northwest China, with 108 counties. It covers an area of 205,624 km^2^, with mountains and loess plateaus constituting 76% of the territory. The altitude varies tremendously, ranging from 170 to 3,767 m. The nominal gross domestic product (GDP) in 2019 was 2.58 trillion RMB (US$ 399.79 billion). The GDP per capita in 2019 was 66,649 RMB (US$ 10,331)^18^, which was lower than the national average^19^.

### Data collection

PTB is listed as a class B notifiable infectious disease in China. Medical institutions are required to report PTB cases to the China Centres for Disease Control and Prevention (CCDC) via the National Notifiable Infectious Diseases Reporting Information System (NIDRS)^16^. PTB cases were diagnosed through pathogen detection, X-rays, and pathologic diagnoses according to the National TB Diagnosis and Treatment Guideline of China. PTB case information from 1 January 2011 to 31 December 2018, including patient home address, date of diagnosis, smear microscopy results, and diagnosis type, was acquired from the Shaanxi Centre for Disease Control and Prevention. Four types of PTB cases were diagnosed: sputum smear-positive PTB, sputum smear-negative PTB, sputum smear-negative but culture-positive PTB, and PTB without a sputum smear test. Notified BP-PTB cases included sputum smear-positive PTB and sputum smear-negative but culture-positive PTB. Since 1 July 2017, rifampicin-resistant tuberculosis (RR-TB) information has been added to the national infectious disease surveillance system; thus, notified BP-PTB in 2017 and 2018 also included RR-TB cases. PTB cases without sputum smear tests mainly refer to patients who had slight symptoms and did not cough up sputum. Population data were obtained from the Shaanxi Statistical Yearbook (2011–2018).

### Diagnostic criteria and quality assurance of BN-PTB

Notified BN-PTB cases refer to clinically diagnosed patients whose sputum smear and culture were both negative. According to the National TB Diagnosis and Treatment Guidelines of China, a case is diagnosed as BN-PTB after other lung diseases have been excluded by differential diagnosis and one of the following criterion is met: (1) abnormalities consistent with active PTB on chest radiography and clinical features of PTB; (2) abnormalities consistent with active PTB on chest radiography and moderately positive or strongly positive tuberculin test; (3) abnormalities consistent with active PTB on chest radiography and positive interferon-gamma release; (4) abnormalities consistent with active PTB on chest radiography and a positive *Mycobacterium tuberculosis* antibody test; or (5) abnormalities consistent with active PTB on chest radiography and TB lesions confirmed by extra-pulmonary histopathological examination^20^.

A range of measures was employed to ensure the diagnostic quality of BN-PTB cases. First, BN-PTB cases were diagnosed and treated according to the National TB Diagnosis and Treatment Guidelines of China, and these diagnostic criteria for BN-PTB are consistent with that of the World Health Organization (WHO)^21^. The diagnosis of BN-PTB was made by a team of physicians from the respiratory, laboratory and radiology departments. Doctors made the diagnosis according to the uniform criteria mentioned above. Therefore, there was generally no bias. Furthermore, specialists in PTB from provincial or municipal hospitals appointed by the provincial centre for disease control and prevention regularly reviewed and assessed medical records of BN-PTB cases diagnosed at county hospitals^13^. The checklist included information about symptoms, diagnosis (including sputum smear, sputum culture, rapid molecular tests, chest X-ray and computed tomography (CT) examinations, immunological examinations, and differential diagnosis information), and treatment regimens. During the monitoring process, misdiagnosis of BN-PTB cases was promptly identified and corrected. Therefore, bias was minimised as much as possible. In addition, PTB cases were notified according to their current address instead of the address of the hospital where they were diagnosed^12^. The geographical distribution of PTB cases was not affected by the location of doctors who attended them.

To protect the privacy of patients, the names of patients were anonymized and numbered. Then, the information of each patient was sorted. The current address information in the PTB case information database was collated, and the county to which each patient's current address belonged was extracted. A total of 149 cases were eliminated due to incomplete or untraceable addresses.

### Data analysis

1. **Descriptive analysis**
  The age-specific incidence rate of notified BP-PTB and notified BN-PTB was calculated. A line chart of monthly cases was constructed to show the temporal distribution of notified BP-PTB cases and notified BN-PTB cases. The annual incidence rates of notified BP-PTB and notified BN-PTB were calculated. Then, the annual incidence was linked to each county on a digital map of Shaanxi to display the spatial variation.
2. **Spatial autocorrelation analysis**
  Moran’s *I *was calculated to examine the spatial autocorrelation of notified BP-PTB and notified BN-PTB in Shaanxi. Moran's *I *is calculated based on the deviation from the means of two neighbouring values^22^. The value of Moran’s *I *ranges between − 1 (the maximum negative association) and 1 (the maximum positive association). A high positive value indicates a strong spatial autocorrelation, and vice versa for a negative value^23^. The significance of the estimated Moran’s *I* was examined by the *Z* test (Monte Carlo randomization, 999 permutations)*.* The PTB incidence was regarded as statistically spatially clustered when the *Z* score was greater than or equal to 1.96^9^. The global Moran’s *I* was calculated to examine the overall spatial autocorrelation. Anselin’s local Moran’s *I* was used to assess the local spatial autocorrelation and determine the locations of the clusters. The high–high (HH), low–low (LL), and outlying local cluster (high–low (HL) and low–high (LH)) locations were visualized with local indicators of a spatial association (LISA) cluster map.
3. **Spatiotemporal cluster analysis**
  To identify geographic areas and time periods with high BP-PTB and BN-PTB incidence, space–time scan statistics for notified BP-PTB cases and notified BN-PTB cases were performed. SaTScan (version 9.4.4, Kulldorff and Information Management Services Inc, MA, USA) was used, and a discrete Poisson regression model was constructed. The scan window was a cylinder, with a circular geographical base and height corresponding to time. The mean relative risks (*RRs*) and log-likelihood ratios (*LLRs*) were calculated to identify the primary cluster and the secondary clusters^24^. The maximum length of the cluster radii was set to 100 km. A similar method of setting cluster radii was used in another spatiotemporal cluster study^25^. The criterion for reporting secondary clusters was no geographical overlap.
  SPSS software (version 18.0, International Business Machines Corporation, NY, USA) was used for data management and demographic characteristic analyses. GeoDa (version 1.12, GeoDa Centre for Geospatial Analysis and Computation, Arizona State University, AZ, USA) was used to create the incidence map, calculate Moran’s *I* and display the spatiotemporal clusters.

### Ethics approval and consent to participate

The research protocol was approved by the Institutional Review Board of Xi’an Jiaotong University Health Science Centre. This study was performed in accordance with the Declaration of Helsinki. PTB data were collected through routine TB surveillance activities. Personal information, such as the patient’s name and identity number, was not required. This study only analysed aggregated data at the county level. Therefore, the need for written informed consent was waived.

## Results

### Demographic characteristics of notified BP-PTB and notified BN-PTB

A total of 180,512 PTB cases were included in this study. The numbers of notified BP-PTB cases and BN-PTB cases were 44,894 and 116,447, respectively. The average notified annual incidence rates of PTB, BP-PTB and BN-PTB were 59.50 per 100,000 persons, 14.80 per 100,000 persons, and 38.38 per 100,000 persons between 2011 and 2018, respectively. The notified incidence rates of BP-PTB and BN-PTB between 2011 and 2018 are presented in Table 1.

**Table 1:** The notified incidence rate of BP-PTB and BN-PTB in Shaanxi (2011–2018).

Year	BP-PTB (1/100,000 persons)	BN-PTB (1/100,000 persons)
2011	25.24	34.61
2012	17.23	38.92
2013	12.88	40.65
2014	9.60	39.82
2015	8.80	39.81
2016	8.72	40.18
2017	13.21	39.35
2018	13.29	33.76
2011–2018	14.80	38.38

**Figure 1:**
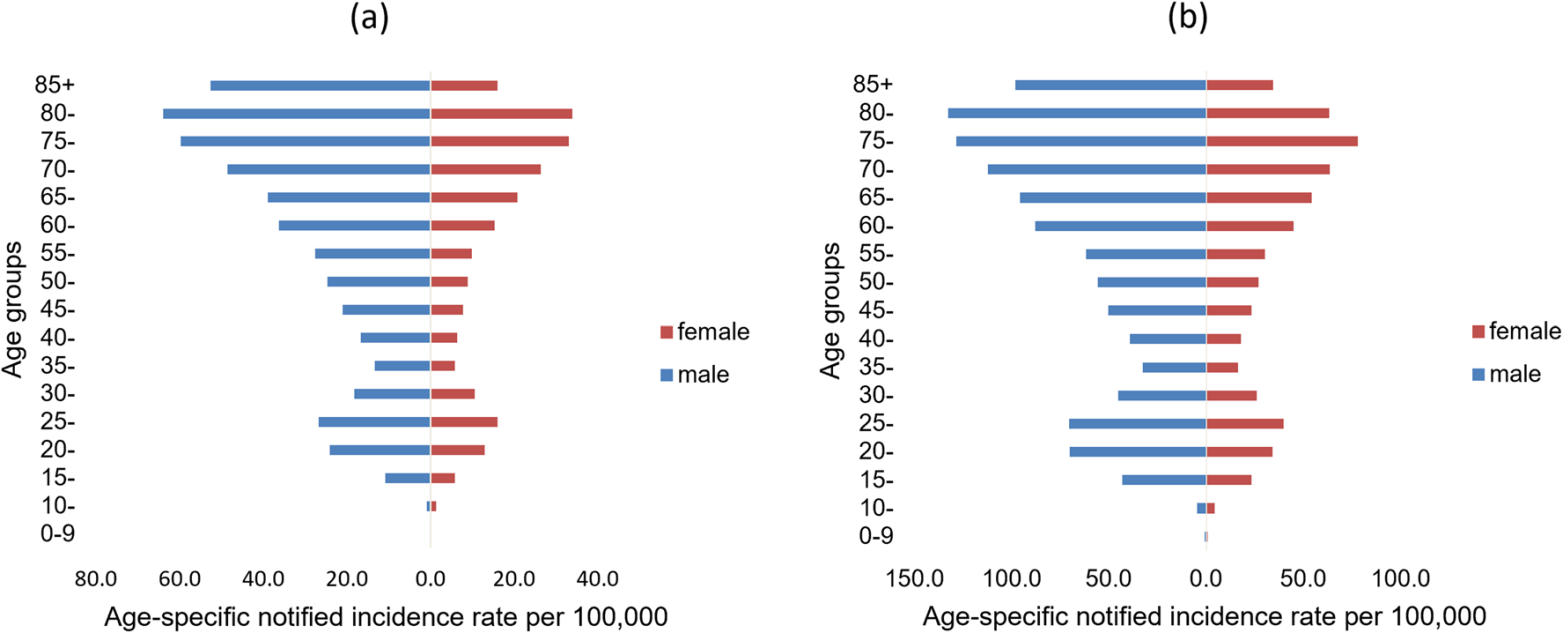
Age-specific notified incidence rate of BP-PTB and BN-PTB.

Both the notified incidence rates of BP-PTB and BN-PTB showed an overall increasing trend with age, peaking in the 80–84-year-old group. The one exception was that the notified peak of BN-PTB for females occurred in the 75–80-year-old group. In addition, the notified incidence rates of BP-PTB and BN-PTB in men were higher than those in women in all age groups (Fig. 1).

### Temporal patterns of notified BP-PTB and notified BN-PTB

**Figure 2:**
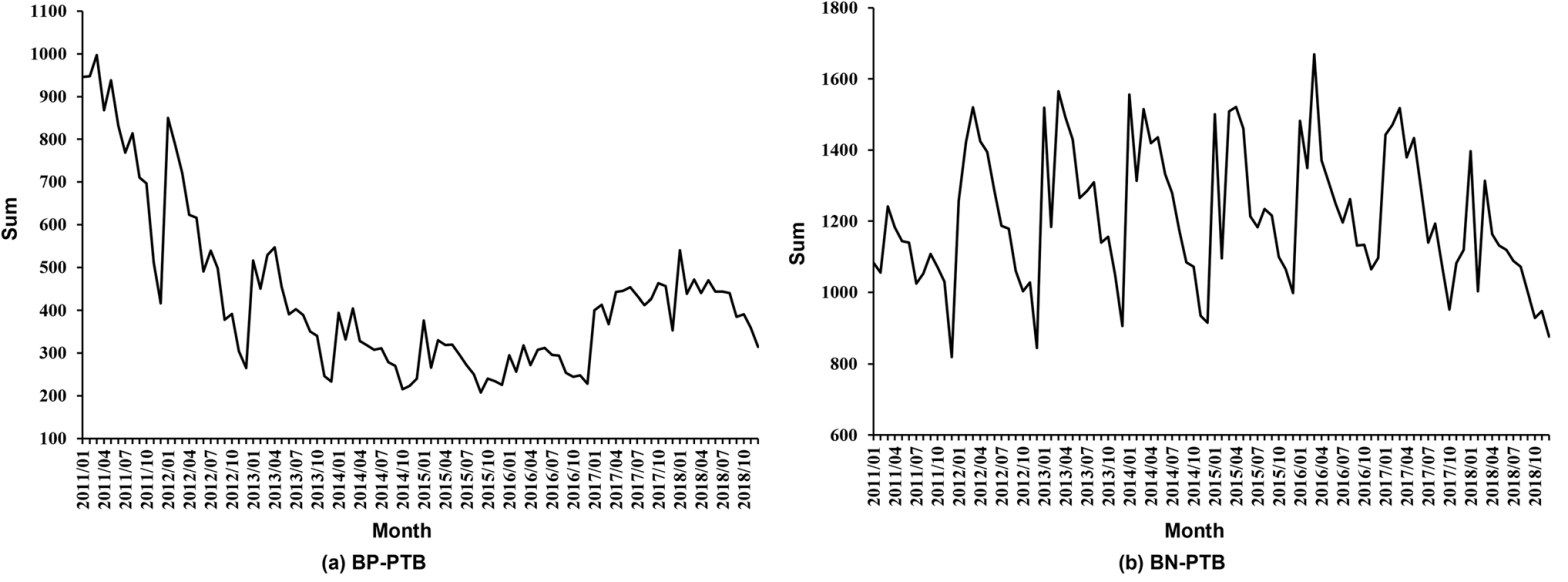
The monthly distribution of BP-PTB cases and BN-PTB cases.

As shown in Fig. 2, from 2011–2016, the notified incidence of BP-PTB demonstrated a decreasing trend. Nevertheless, a significant upwards trend was noted after 2017 with a peak in January 2018. In most years, the highest number of BN-PTB cases was notified between January and March, whereas the lowest was notified between November and December. Compared with that of January and March, the number of notified BN-PTB cases was lower in February. The number of notified BP-PTB cases showed similar seasonal characteristics. Except in 2011, 2012, and 2017, the number of notified BP-PTB cases decreased in February and then increased in March compared with that of January (Fig. 2).

### Spatial distribution characteristics of the notified incidence rates of BP-PTB and BN-PTB

**Figure 3:**
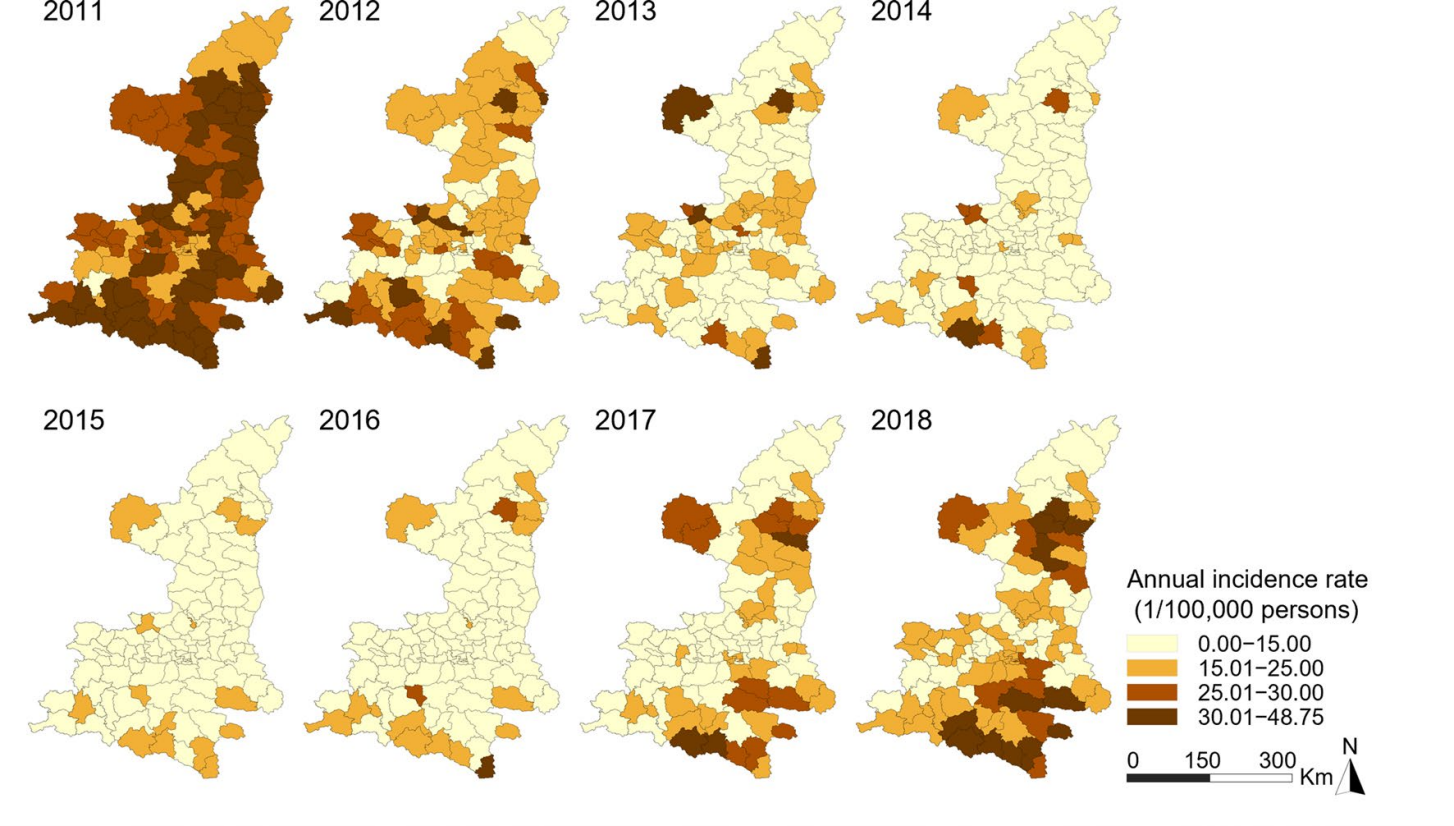
Annual notified incidence rate of BP-PTB.

**Figure 4:**
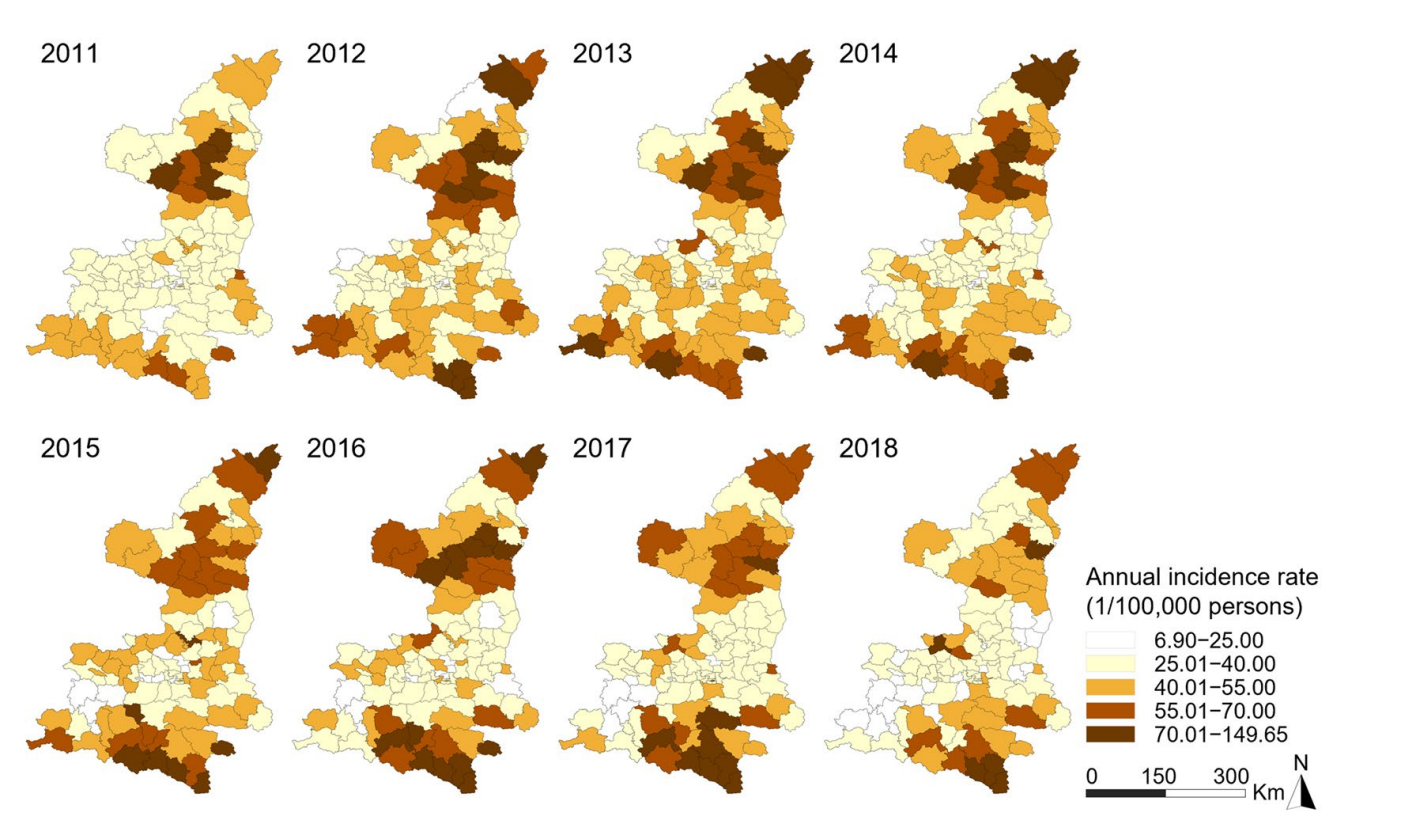
Annual notified incidence rate of BN-PTB.

The spatial distributions of the notified incidence rates of BP-PTB and BN-PTB cases at the county level are shown in Figs. 3 and 4, respectively. During the study period, the notified BP-PTB epidemic was at its worst in 2011, followed by a decline from 2012 to 2015 and an increase after 2016. The notified BP-PTB epidemic decreased rapidly in the central region. The increasing trend of the notified BP-PTB epidemic was more pronounced in the southern and northern regions than in the central region after 2016. The spatial distribution of the average annual incidence rate of BP-PTB is shown in Supplementary Fig. S1a. The notified incidence rate of BN-PTB increased obviously from 2011 to 2014 and then decreased slowly in the north. It is worth noting that the notified incidence rate of BN-PTB showed a consistent increasing trend in the south. The notified BN-PTB epidemic increased slowly and then decreased rapidly in the central region. The spatial distribution of the average annual incidence rate of BN-PTB is shown in Supplementary Fig. S1b.

### Spatial autocorrelation patterns of the notified incidence rate of BP-PTB and BN-PTB

Positive autocorrelations (global Moran’s *I* > 0) in the notified incidence rate of BP-PTB and BN-PTB were identified between 2011 and 2018 (P < 0.05, Table 2). The spatial autocorrelations in the notified incidence rate of BP-PTB did not show a significant increasing trend (r = 0.599, P > 0.05). The spatial autocorrelations in the notified incidence rate of BN-PTB showed significant temporal variation (r = 0.857, P < 0.05), which suggested significant escalated regional differences in the incidence of notified BN-PTB.

**Table 2:** Global spatial autocorrelations of the notified incidence rate of BP-PTB and BN-PTB in Shaanxi (2011–2018).

Year	BP-PTB			BN-PTB		
	Moran’s *I*	*Z*	*P*	Moran’s *I*	*Z*	*P*
2011	0.27	4.40	<0.001	0.39	6.82	<0.001
2012	0.23	3.74	<0.001	0.49	8.22	<0.001
2013	0.19	3.17	<0.001	0.43	7.48	<0.001
2014	0.21	3.52	<0.001	0.45	7.90	<0.001
2015	0.27	4.38	<0.001	0.52	8.47	<0.001
2016	0.27	4.39	<0.001	0.63	10.42	<0.001
2017	0.36	5.66	<0.001	0.69	11.57	<0.001
2018	0.49	7.76	<0.001	0.53	9.14	<0.001
2011–2018	0.39	6.22	<0.001	0.65	10.76	<0.001

**Figure 5:**
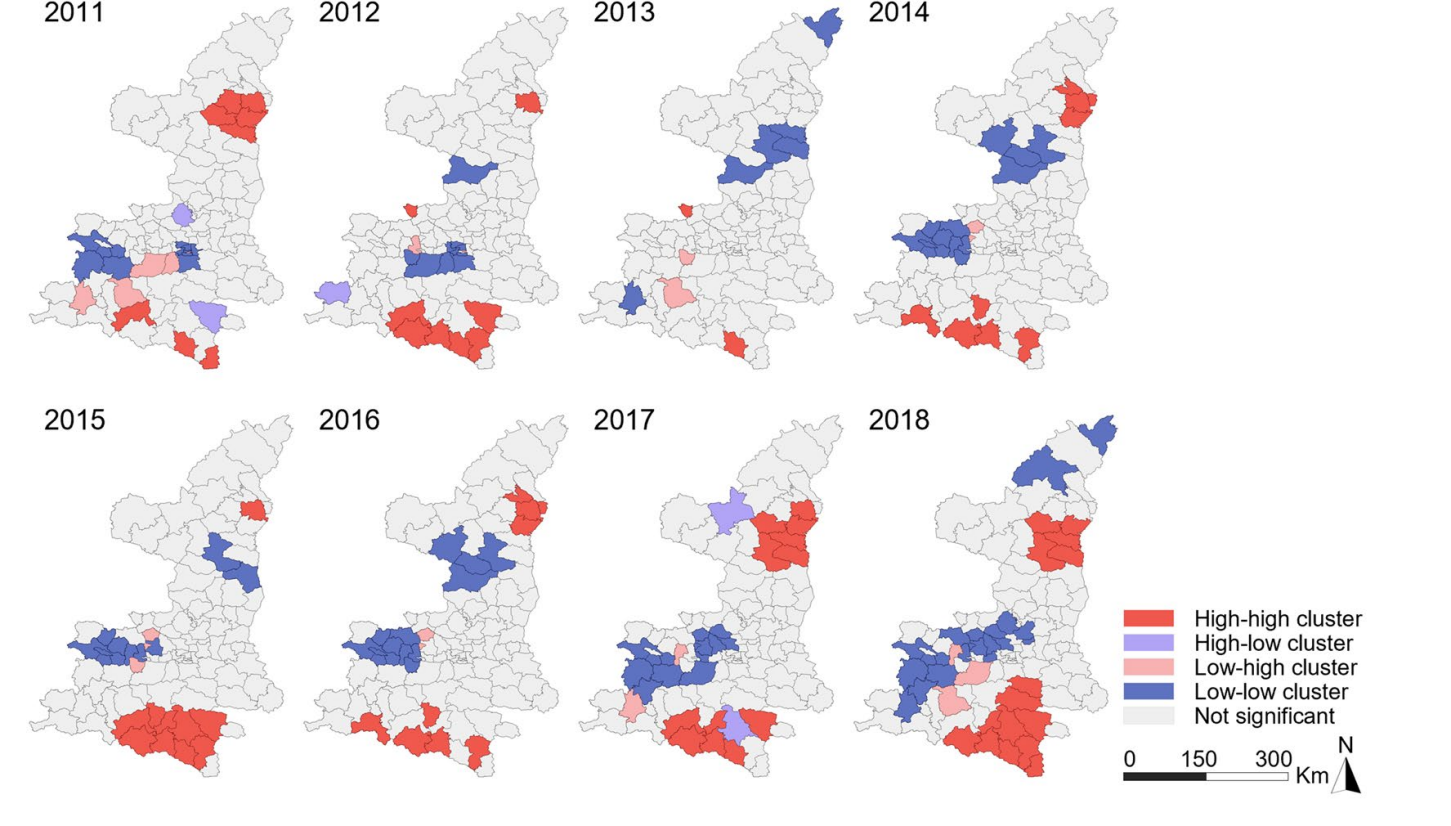
LISA cluster map for BP-PTB.

**Figure 6:**
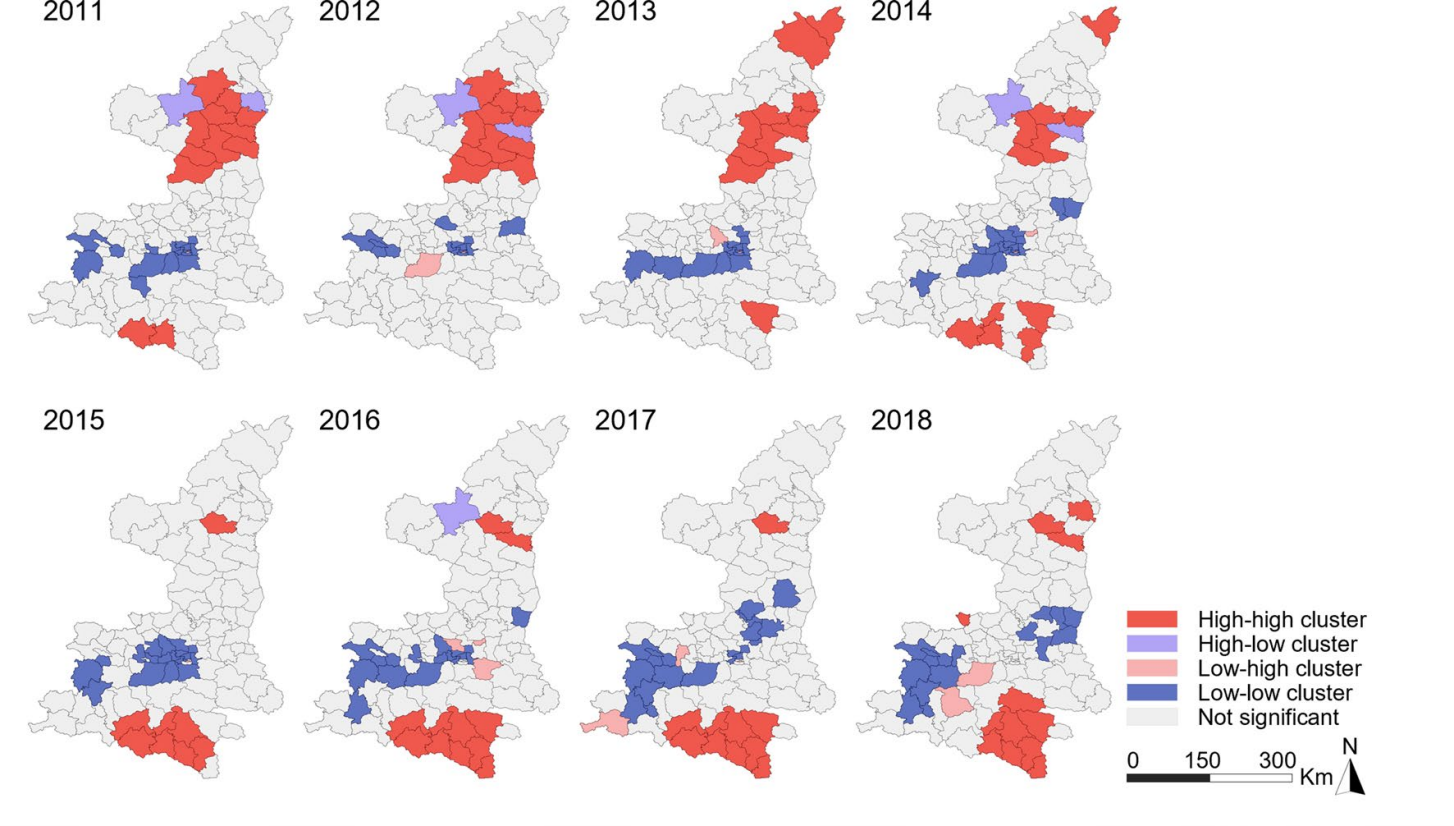
LISA cluster map for BN-PTB.

The results of Anselin’s local Moran’s *I* analysis showed that the southernmost region exhibited an HH correlation for the notified incidence rate of BP-PTB (Fig. 5). The number of counties in the southernmost region exhibiting HH clusters of notified BP-PTB increased from 3 in 2011 to 10 in 2018. The HH clusters of notified BN-PTB were mainly located in the north between 2011 and 2014, while most of them were located in the south after 2014 (Fig. 6). The central region exhibited an LL correlation for both notified BP-PTB and notified BN-PTB. Few significant spatial outliers (LH and HL clusters) were observed. Anselin’s local Moran’s *I* values of the average annual incidence rates of notified BP-PTB and BN-PTB are shown in Supplementary Fig. S2a and Supplementary Fig. S2b, respectively.

**Figure 7:**
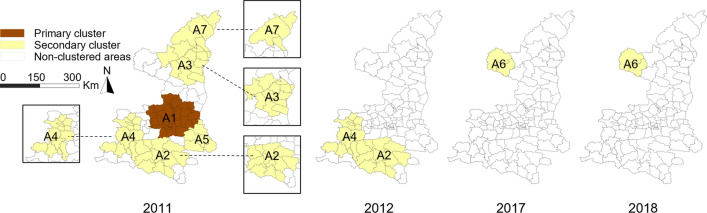
Spatiotemporal clusters of BP-PTB.

The space–time scan statistics for notified BP-PTB cases identified seven significant clusters (Fig. 7) over the study period. The *RR* of acquiring BP-PTB infection inside the clusters to that outside the clusters ranged from 1.45 to 2.09. The primary cluster (Cluster A1) was located in the central plain in 2011. It involved thirty-nine counties and 4,885 cases, with an *RR* of 1.77. The coordinates of the cluster centre are 34°71'N and 108°94'E. Cluster A2 was mainly distributed in the middle of the southern region from 2011 to 2012. This cluster included 2,077 cases, with an *RR* of 1.94. Other clusters were located in the northern, southwestern and southeastern regions (Supplementary Table S1).

**Figure 8:**
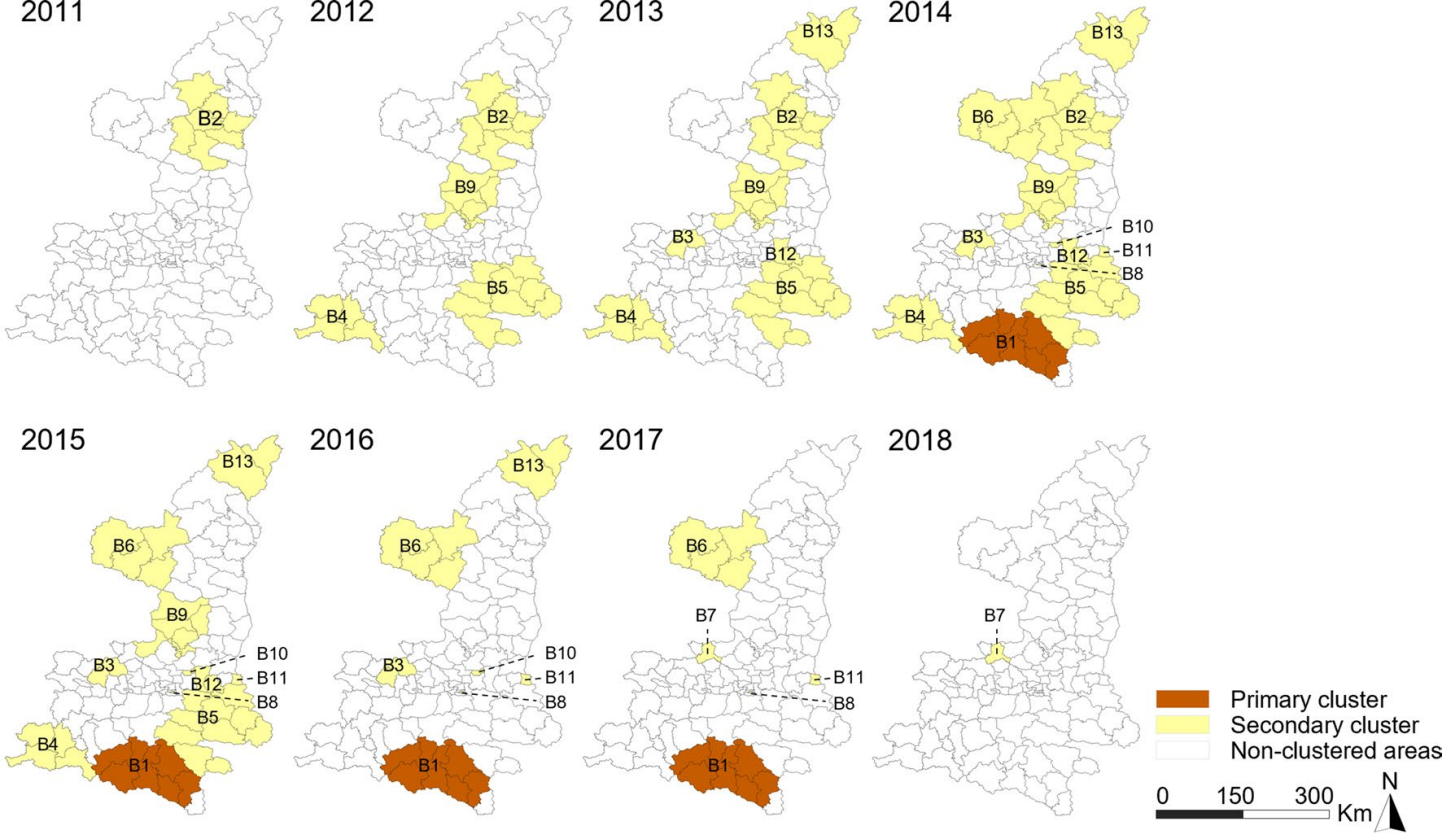
Spatiotemporal clusters of BN-PTB.

The space–time scan statistics for notified BN-PTB cases identified 13 significant clusters (Fig. 8). The primary cluster of notified BN-PTB cases (cluster B1) involved eight counties and was located in the mountainous areas of the southernmost region. It included 7,293 cases and persisted for a long time, from 2014 to 2017, with an RR of 1.85. The coordinates of the cluster centre are 32°48'N and 108°40'E. Cluster B2 was located in the eastern region of the north from 2011 to 2014 with an *RR* of 1.74. Cluster B6 was located at the northwest border of Shaanxi. This cluster included 2,016 cases with an RR of 1.27. Cluster B8 was located in the central urban district of the provincial capital city (Beilin district, Xi’an City). This cluster occurred from 2014 to 2017, with an *RR* of 1.33. More details on the other clusters are displayed in Table S2.

## Discussion

This study explored the spatiotemporal patterns of notified BP-PTB and BN-PTB cases using advanced spatiotemporal statistics in Shaanxi, Northwest China. Clusters of both notified BP-PTB and BN-PTB were located in the mountainous areas in the southernmost region. This study also found that the primary spatiotemporal clusters of notified BP-PTB and BN-PTB were located in different regions. This difference was partly due to an early detection policy. Our study provides evidence for intensifying PTB control activities in these geographical clusters.

The fluctuations in the incidence rate of BP-PTB were caused by the early detection policy and the application of advanced testing techniques. The early detection policy established in the twelfth Five-Year (2011–2015) National TB Integrated Control Plan, which was initiated in 2011, facilitated the detection of BP-PTB^26^. The implementation of this policy and subsequent interventions were effective, and therefore the incidence rate decreased between 2012 and 2016. The significant upwards trend in the BP-PTB incidence after 2017 was attributable to improved laboratory testing capacity, which is mainly due to the availability of molecular detection techniques. The proportion of designated hospitals capable of performing sputum culture and those performing Xpert *Mycobacterium tuberculosis*/rifampicin (MTB/RIF) assays were 55.56% (60/108) and 49.07% (53/108) in 2017, respectively, compared to 12.04% (13/108) and 5.56% (6/108) in 2014, respectively^27^. Other molecular detection techniques, such as loop-mediated isothermal amplification (LAMP) and cross-priming amplification (CPA), were also applied. The molecular detection techniques are more accurate than traditional sputum smears in diagnosing BP-PTB^28,29^, thus increasing the rate of BP-PTB cases detected. Generally, January was one of the months with the highest number of notified BP-PTB cases. Given the impact of improved detection techniques, a peak in patients occurred in January 2018.

The notification peaks of BP-PTB and BN-PTB mainly occurred in March. Similar temporal trends have been reported in other studies^30,31^. Vitamin D deficiency due to reduced sunlight exposure, increased transmission due to household crowding and poorer ventilation in winter could increase PTB infection^32,33^. In chronic infectious diseases, time is needed for the symptoms to develop and become evident. In addition, there is a delay between the onset of symptoms in patients and the care-seeking period^34^. Moreover, avoiding healthcare appointments during the Chinese Lunar New Year festival is traditional and likely contributed to the lower number of reported cases in February compared with that in January and March. Furthermore, undetected cases persisting from February may increase the number of cases identified in March. All these factors contribute to the notification peak that occurs in early spring.

In every year, the global Moran's *I* was higher in notified BN-PTB cases than in BP-PTB cases, indicating that the spatial autocorrelation of BN-PTB was stronger than that of BP-PTB. Wubuli also found a similar phenomenon; specifically, Moran’s *I* of smear-negative PTB was higher than that of smear-positive PTB between 2011 and 2013 in Xinjiang, China^16^. Furthermore, the global Moran’s *I* of BN-PTB showed a significant increasing trend, which indicated escalated regional differences for BN-PTB. The expanded LL clusters indicated effective BN-PTB control. However, the HH clusters of notified BN-PTB remained relatively stable, indicating that it is difficult to control the BN-PTB epidemic in these areas. Therefore, the HH clusters should be considered priority regions in the future.

The early detection policy in 2011 facilitated detection of the primary cluster (Cluster A1) for notified BP-PTB. Cluster A1 was located in a well-developed region with a high population density. Case detection was improved due to advanced healthcare services and adequate human resources, thus forming the primary cluster. A study in Yunnan also suggested that early case identification during the implementation of the national PTB control plan facilitated BP-PTB case detection^26^. The primary cluster of notified BN-PTB cases was characterized by relatively low socioeconomic status and limited geographic access to health care. This cluster was located deep in the mountainous areas, and five of the eight counties were considered high-poverty counties. In addition, Cluster B2 was located in the eastern region of the north. This area is more mountainous and experienced slower economic development than the western region of the north, which may have led to it becoming a high-risk area. TB is common in poor and deprived populations^35,36^. Barriers to public transportation and care deep in the mountains also delayed care seeking and diagnosis^37^. Efforts to mitigate poverty and enhance healthcare services, as well as measures to improve living conditions, should be promoted to eliminate TB^38^.

Due to changes in PTB control policies and the application of advanced testing techniques, the spatial and temporal characteristics of BP-PTB and BN-PTB appear inconsistent. However, the second cluster of BP-PTB (Cluster A2) was located in the mountainous areas of the southernmost region. Thus, the cluster location was mostly consistent with the primary BN-PTB cluster. According to the different spatial analyses, the high epidemic rates of BP-PTB and BN-PTB in the southernmost region and the eastern region of the north were stable during the study period.

Easy access to public transportation and prosperous trade facilitate contact with infectious patients and thus favour transmission across provincial boundaries. Located in a hub of commercial activities, Cluster B6 bordered three provinces with a high TB burden. Individuals frequently crossed the border from these provinces to Shaanxi for economic and social reasons, which could contribute to maintenance of the long-term epidemic^39,40^.

Cluster B8 (Beilin district) occurred in a major business centre with a high education base in Xi’an city, the provincial capital. High infection rates among migrants and college students contributed to this cluster. There were approximately 12,000 college students per year in the cluster. The proportion of college students in the cluster was 14%, which was almost twice the average proportion (7.19%) of college students among the entire population in Xi’an city. Other clusters that lasted for short periods, from one month to four months, were associated with occasional screenings or active case detections^41^.

Overall, both notified BP-PTB and BN-PTB epidemics in the northern region showed a decreasing trend during the study period. The following facts may explain these changes. First, the rapid economic development in the northern region, especially in the northwest, has led to a decline in the epidemic due to the improvement of living standards and better housing and nutritional conditions^38^. Along with the improved economic situation, people have become more aware of health care and are more likely to take the initiative to seek medical treatment for their illnesses, which leads to early detection and treatment, thus reducing the spread of PTB. Second, because the epidemic in the northern region has been more serious, PTB control has received increasing attention by the local government, including PTB screening for key groups such as elderly individuals and close contacts of PTB patients and students^12^. The local government also provides financial support for PTB screening and treatment. All these PTB prevention and treatment efforts contributed to the decline in PTB incidence in the north.

This was the first case study to explore the spatiotemporal patterns of notified BP-PTB and BN-PTB at the county level in Northwest China. Multiple spatial cluster analyses were explored to determine the cluster times, locations, and relative risks of high-risk areas. Poor mountainous areas have a heavy burden of notified BP-PTB and BN-PTB, and these individuals have poor access to health services. Therefore, the intensification of PTB control activities among high-risk populations in this area should receive more attention.

This study also had some limitations. First, overdiagnosis or misdiagnosis of BN-PTB is an issue. However, due to the lack of an aetiological gold standard, it is difficult to avoid the overdiagnosis or misdiagnosis of BN-PTB. Second, our results indicated that socioeconomic development and the application of molecular detection techniques may be related to the notified incidence rate of BP-PTB and BN-PTB. The associations between various ecological factors and PTB clusters will be explored in future studies. Third, BP-PTB and BN-PTB incidences may be underestimated because this study did not include cases that were not reported in the official system. Fourth, the cylindrical window of the space–time scan statistics (with a circular spatial base) did not allow for irregular cluster shapes.

## Conclusions

This study reported notable spatial and temporal heterogeneity in notified BP-PTB and BN-PTB incidences across Shaanxi, Northwest China. The primary cluster of notified BP-PTB was located in the central plain in 2011. The primary cluster of notified BN-PTB was located in the mountainous areas of the southernmost region from 2014 to 2017. Our study provides evidence for intensifying PTB control activities in these geographical clusters. Further study is needed to explore the association between various ecological factors and the detected spatiotemporal distribution of BP-PTB and BN-PTB.

## Supplementary Information

Supplementary Information 1.

Supplementary Information 2.

Supplementary Information 3.

## Abbreviations

PTB: Pulmonary tuberculosis
TB: Tuberculosis
BP-PTB: Bacteriologically positive PTB
BN-PTB: Bacteriologically negative PTB
CCDC: Chinese Centre for Disease Control and Prevention
NIDRS: National notifiable infectious diseases reporting information system
HH: High–high
LL: Low–low
HL: High–low
LH: Low–high
LISA: Local indicators of spatial association
*RR*: Relative risk
*LLR*: Log-likelihood ratio
