# Supplementary figures and images for: Distribution of bacteriologically positive and bacteriologically negative pulmonary tuberculosis in Northwest China: spatiotemporal analysis
Source: Sci Rep. 2022 Apr 27;12:6895. doi: 10.1038/s41598-022-10675-1 (PMC9046232; doi:10.1038/s41598-022-10675-1)

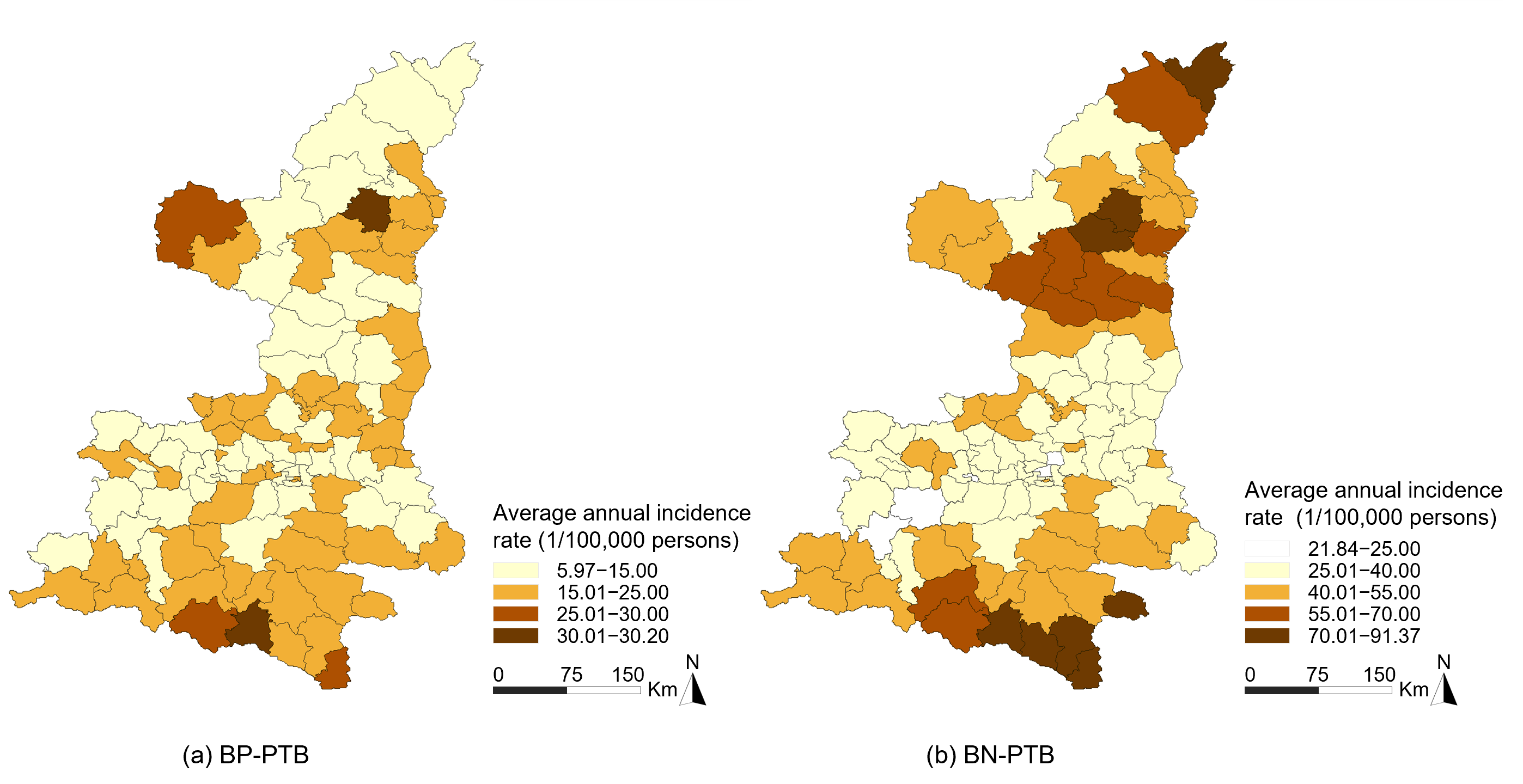

Supplement: Supplementary file 1 — Supplementary Information 1. [file 41598_2022_10675_MOESM1_ESM.png]

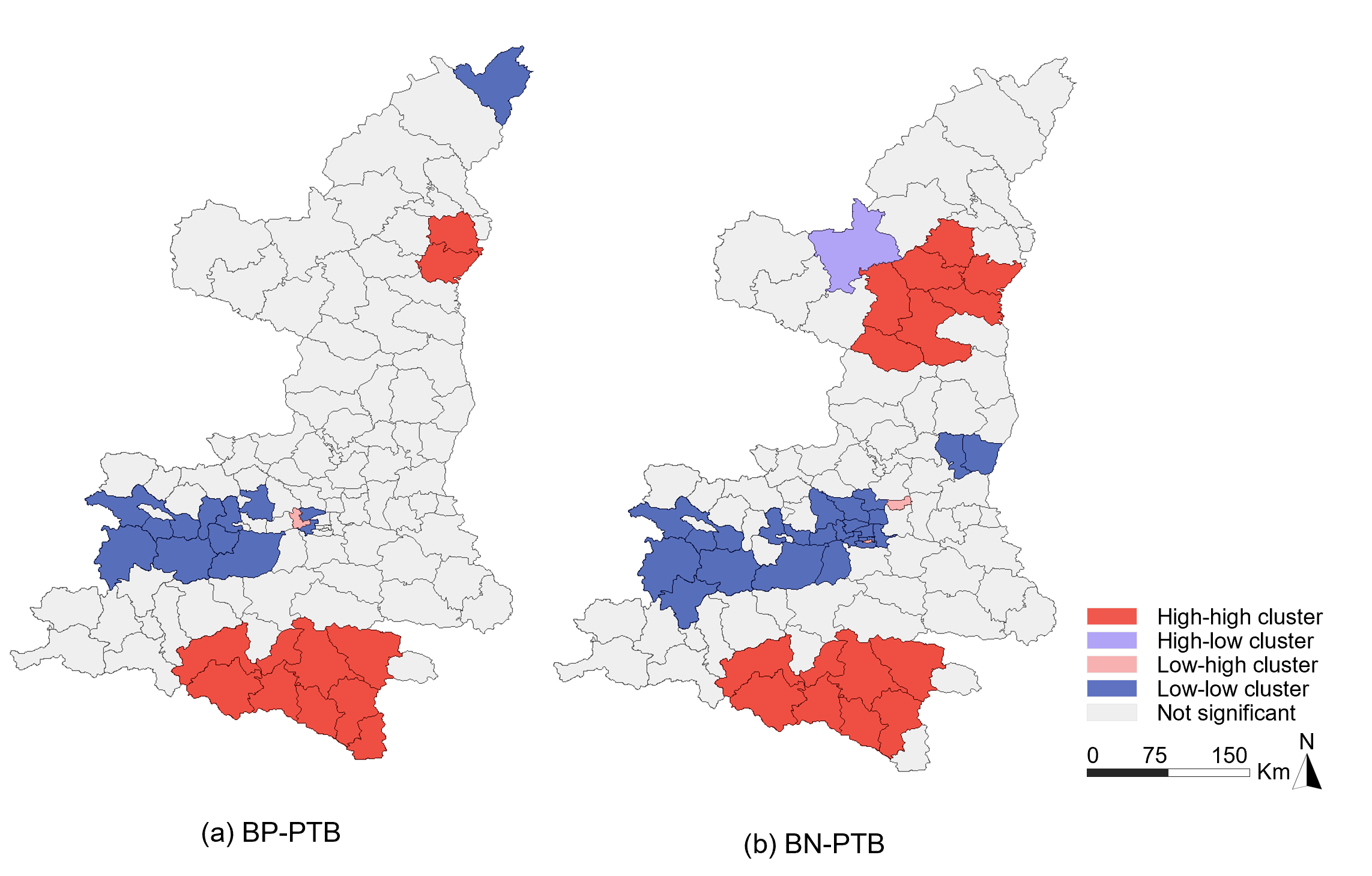

Supplement: Supplementary file 2 — Supplementary Information 2. [file 41598_2022_10675_MOESM2_ESM.png]
