# Supplementary material for: Distribution of bacteriologically positive and bacteriologically negative pulmonary tuberculosis in Northwest China: spatiotemporal analysis
Source: Sci Rep. 2022 Apr 27;12:6895. doi: 10.1038/s41598-022-10675-1 (PMC9046232; doi:10.1038/s41598-022-10675-1)
Supplement: Supplementary file 3 — Supplementary Information 3. [file 41598_2022_10675_MOESM3_ESM.docx]

**Supplementary material**

Table S1: Spatiotemporal clusters of BP-PTB cases in Shaanxi (2011- 2018)

| Type | Cluster number  in figure | Coordinates/Radius | N | Time frame | Population | Observed cases | Expected cases | Annual incidence rate  (1/100,000 persons) | *RR* | *LLR* | *P* |
| --- | --- | --- | --- | --- | --- | --- | --- | --- | --- | --- | --- |
| Primary | A1 | (34.71 N，108.98 E) / 100 km | 39 | 2011 | 18,494,600 | 4885 | 2892 | 26.41 | 1.77(1.72-1.82) | 612.52 | <0.001 |
| Secondary | A2 | (32.90 N，108.46 E) / 94 km | 12 | 2011−2012 | 7,005,739 | 2077 | 1097 | 29.65 | 1.94(1.85-2.02) | 356.44 | <0.001 |
|  | A3 | (37.25 N，109.62 E) / 99 km | 12 | 2011 | 2,635,100 | 855 | 412 | 32.45 | 2.09(1.96-2.24) | 183.33 | <0.001 |
|  | A4 | (33.94 N，106.77 E) / 98 km | 12 | 2011−2012 | 7,688,863 | 1847 | 1204 | 24.02 | 1.56(1.49-1.63) | 151.97 | <0.001 |
|  | A5 | (33.66 N，110.47 E) / 97 km | 6 | 2011 | 2,063,900 | 584 | 323 | 28.30 | 1.82(1.68-1.97) | 85.81 | <0.001 |
|  | A6 | (37.35 N，107.82 E) / 47.91 km | 2 | 2017-2018 | 971,597 | 270 | 152 | 27.79 | 1.78(1.58-2.01) | 37.47 | <0.001 |
|  | A7 | (38.84 N，110.30 E) / 89 km | 4 | 2011 | 1,559,200 | 353 | 244 | 22.64 | 1.45(1.31-1.61) | 21.20 | <0.001 |

Table S2: Spatiotemporal clusters of BN-PTB cases in Shaanxi (2011- 2018)

| Type | Cluster number  in figure | Coordinates/Radius | N | Time frame | Population | Observed  cases | Expected  cases | Annual incidence rate  (1/100,000 persons) | *RR* | *LLR* | *P* |
| --- | --- | --- | --- | --- | --- | --- | --- | --- | --- | --- | --- |
| Primary | B1 | (32.48 N，108.40 E) / 86 km | 8 | 2014−2017 | 10,110,363 | 7293 | 4058 | 72.13 | 1.85（1.80-1.89） | 1084.59 | < 0.001 |
| Secondary | B2 | (37.25 N，109.62 E) / 71 km | 7 | 2011−2014 | 6,524,700 | 4486 | 2619 | 68.75 | 1.74（1.69-1.79） | 561.61 | < 0.001 |
|  | B3 | (38.84 N，110.30 E) / 56 km | 2 | 2013−2016 | 2,895,700 | 2390 | 1163 | 82.54 | 2.08（2.00-2.16） | 501.15 | < 0.001 |
|  | B4 | (32.92 N，106.04 E) / 86 km | 4 | 2012−2015 | 5,494,018 | 3011 | 2205 | 54.81 | 1.38（1.33-1.43） | 134.63 | < 0.001 |
|  | B5 | (33.44 N，110.01 E) / 88 km | 10 | 2012−2015 | 13,854,352 | 6395 | 5562 | 46.16 | 1.16（1.13-1.19） | 62.50 | < 0.001 |
|  | B6 | (37.35 N，107.82 E) / 96 km | 4 | 2014−2017 | 3,963,389 | 2016 | 1591 | 50.87 | 1.27（1.21-1.33） | 52.93 | < 0.001 |
|  | B7 | (35.07 N，108.09 E) / 0 km | 1 | 2017−2018 | 659,801 | 447 | 265 | 67.75 | 1.69（1.54-1.86） | 52.08 | < 0.001 |
|  | B8 | (34.25 N，108.95 E) / 0 km | 1 | 2014−2017 | 2,543,400 | 1349 | 1021 | 53.04 | 1.33（1.26-1.40） | 48.14 | < 0.001 |
|  | B9 | (35.59 N，108.99 E) / 58 km | 7 | 2012−2015 | 5,141,705 | 2503 | 2064 | 48.68 | 1.22（1.17-1.27） | 44.47 | < 0.001 |
|  | B10 | (34.67 N，109.29 E) / 0 km | 1 | 2014−2016 | 864,500 | 470 | 347 | 54.37 | 1.36（1.24-1.48） | 19.58 | < 0.001 |
|  | B11 | (34.52 N，110.30 E) / 0 km | 1 | 2014−2017 | 634,799 | 358 | 255 | 56.40 | 1.41（1.27-1.56） | 18.57 | < 0.001 |
|  | B12 | (34.53 N，109.58 E) / 25 km | 2 | 2013−2015 | 3,651,900 | 1700 | 1465 | 46.55 | 1.16（1.11-1.22） | 18.14 | < 0.001 |
|  | B13 | (34.77 N，107.68 E) / 33 km | 2 | 2013−2016 | 2,323,317 | 1092 | 933 | 47.00 | 1.17（1.11-1.25） | 13.00 | 0.004 |
